# Imaging the Raf–MEK–ERK Signaling Cascade in Living Cells

**DOI:** 10.3390/ijms251910587

**Published:** 2024-10-01

**Authors:** Young-Chul Shin, Minkyung Cho, Jung Me Hwang, Kyungjae Myung, Hee-Seok Kweon, Zee-Won Lee, Hyun-A. Seong, Kyung-Bok Lee

**Affiliations:** 1 Department of Biochemistry, School of Life Science, Chungbuk National University, Cheongju 28644, Republic of Korea; cubic072@daum.net (Y.-C.S.); tin1400@gmail.com (M.C.); 2 bHLBIO, Cheongju 28119, Republic of Korea; ezone@bhlbio.com; 3 Center for Genomic Integrity, Institute for Basic Science (IBS), Ulsan 44919, Republic of Korea; hjm072@ibs.re.kr (J.M.H.); kjmyung@unist.ac.kr (K.M.); 4 Department of Biomedical Engineering, Ulsan National Institute of Science and Technology (UNIST), Ulsan 44919, Republic of Korea; 5 Center for Bio-Imaging & Translational Research and Bioimaging Data Curation Center, Korea Basic Science Institute (KBSI), Cheongju 28119, Republic of Korea; hskweon@kbsi.re.kr

**Keywords:** ERK pathway, Raf–MEK–ERK signaling cascade, scaffold protein, visualizing protein interaction, cell-based assay

## Abstract

Conventional biochemical methods for studying cellular signaling cascades have relied on destructive cell disruption. In contrast, the live cell imaging of fluorescent-tagged transfected proteins offers a non-invasive approach to understanding signal transduction events. One strategy involves monitoring the phosphorylation-dependent shuttling of a fluorescent-labeled kinase between the nucleus and cytoplasm using nuclear localization, export signals, or both. In this paper, we introduce a simple method to visualize intracellular signal transduction in live cells by exploring the translocation properties of PKC from the cytoplasm to the membrane. We fused bait protein to PKC, allowing the bait (RFP-labeled) and target (GFP-labeled) proteins to co-translocate from the cytoplasm to the membrane. However, in non-interacting protein pairs, only the bait protein was translocated to the plasma membrane. To verify our approach, we examined the Raf–MEK–ERK signaling cascade (ERK pathway). We successfully visualized direct Raf1/MEK2 interaction and the KSR1-containing ternary complex (Raf1/MEK2/KSR1). However, the interaction between MEK and ERK was dependent on the presence of the KSR1 scaffold protein under our experimental conditions.

## 1. Introduction

The mitogen-activated protein kinase (MAPK) pathway is a key signaling pathway that regulates a wide variety of cellular processes, including cell proliferation, survival, differentiation, development, transformation, stress responses, metabolism, and apoptosis. In mammalian cells, three MAPK families have been characterized: extracellular signal-regulated kinase (ERK), C-Jun N-terminal kinase (JNK), and p38 kinase [1,2]. In particular, the Raf–MEK–ERK signaling cascade, often referred to as the ERK pathway, is a well-characterized MAPK pathway. The three-layered MAPK signaling cascade is initiated upon receptor tyrosine kinase (RTK) and Ras activation [3]. For example, many receptors induce the activation of Ras, a small GTPase that binds to GTP, allowing Ras to interact directly with its target proteins (including Raf kinase). Upon its activation, Ras recruits cytosolic Raf to the plasma membrane. This interaction triggers Raf activation, initiating the signal cascade downstream to MEK and ERK.

In addition, scaffold proteins are essential regulators of many key signaling pathways. Despite their diverse functions, they commonly interact and bind with multiple pathway components to form complexes. By organizing these complexes, scaffolds control signal transduction and facilitate the localization of pathway components to specific cellular regions. Several scaffold proteins have also been found to be integral in ERK pathway activation and signaling [4,5,6,7]. One of the best-studied ERK pathway scaffolds, the kinase suppressor of Ras1 (KSR1), was initially identified as a key component in ERK signaling. Induced by epidermal growth factor (EGF), KSR1 translocates to the plasma membrane, where it activates ERK [8,9]. KSR1 has emerged as a major facilitator of the ERK cascade by binding all three kinases in the pathway (Raf, MEK, and ERK). KSR1 not only binds to these kinases but also regulates their activation. For instance, MEK binding to KSR1 stimulates its binding to Raf, resulting in the allosteric activation of Raf kinase activity toward MEK. Similarly, KSR1 preferentially binds to ERK and directs it to cytosolic substrates [10,11].

Fluorescence imaging technology has enabled a new approach to studying intracellular signal transduction, allowing for the analysis of biomolecule behavior in live cells under physiological conditions. Fluorescent biosensors have been remarkably improved in recent years, making them indispensable tools for studying intracellular signal transduction due to their ability to monitor signaling dynamics in real time within living cells [12,13,14]. Fluorescent biosensor imaging techniques such as fluorescence resonance energy transfer (FRET), bimolecular fluorescence complementation (BiFC), and translocation-based biosensors have been developed to analyze cellular signaling and behavior in live cells [15,16,17]. Although obtaining robust FRET and BiFC signals, these techniques require a tedious optimization procedure to determine the relative locations of fluorophores and binding pairs as well as appropriate linker domains.

Translocation-based biosensors (redistribution approach), a cell-based assay technique utilizing protein movement as the readout, have been applied for studying cellular signaling pathways, protein–protein interactions (PPIs), and other intracellular events [18,19,20]. These methods employ a fluorescent-tagged protein that relocates within the cell upon stimulation. Translocation-based cellular assays are robust, fast, and flexible, with high signal-to-noise ratios and minimal construct optimization requirements, making them ideal for high-content drug screening. Despite these advantages, most reported experimental applications have focused on regulated transport between the cell nucleus and the cytoplasm using nuclear localization, export signals, or both [2,15,21].

In a previous study, we developed a translocation-based cellular assay to visualize PPI and its inhibition. The assay relies on the membrane translocation property of protein kinase C (PKC). PKC is widely known to translocate from the cytoplasm to the plasma membrane in response to physiological stimuli and exogenous ligands such as phorbol esters (e.g., phorbol 12-myristate 13-acetate (PMA)). The bait protein was fused to PKCδ, allowing both the bait and target proteins to co-translocate from the cytoplasm to the membrane. In contrast, only the bait protein was translocated to the plasma membrane when chemical inhibitors inhibited bait/target interaction [22,23].

In this study, we expanded the capabilities of our developed translocation-based cellular assay to image intracellular signal transduction. Given its essential role in most RTK functions, the Raf–MEK–ERK signaling cascade was chosen. We initially analyzed the interaction between Raf and MEK, in addition to the interaction between MEK and ERK. However, we did not observe a direct interaction between MEK and ERK. To visualize the complete ERK pathway, we introduced the scaffold protein KSR1, known to interact with both MEK and ERK, enabling visualization of the entire pathway.

## 2. Results and Discussion

The results of a study involving the use of a green fluorescent protein (GFP)-tagged PKC revealed that the dynamics of PKC translocation from the cytoplasm to the plasma membrane in response to different stimuli can be monitored in real-time in live cells [24]. PKC commonly consists of an N-terminal regulatory and C-terminal catalytic (kinase) domain. The regulatory region is divided into an autoinhibitory domain (pseudosubstrate) and typically includes one or two membrane-targeting domains, such as C1 and C2. These domains often bind to diacylglycerol (DAG) and Ca^2+^, respectively; however, their specificities can vary. The novel PKC subfamily (δ, ε, η, θ) includes a C1 domain that binds to DAG but an impaired C2 domain that does not bind to Ca^2+^. Consequently, this subfamily responds to cellular DAG increases but is insensitive to Ca^2+^ [25,26]. Therefore, we used a PKCδ fused to bait protein to co-translocate the target protein from the cytoplasm to the plasma membrane [22,23].

**Figure 1:**
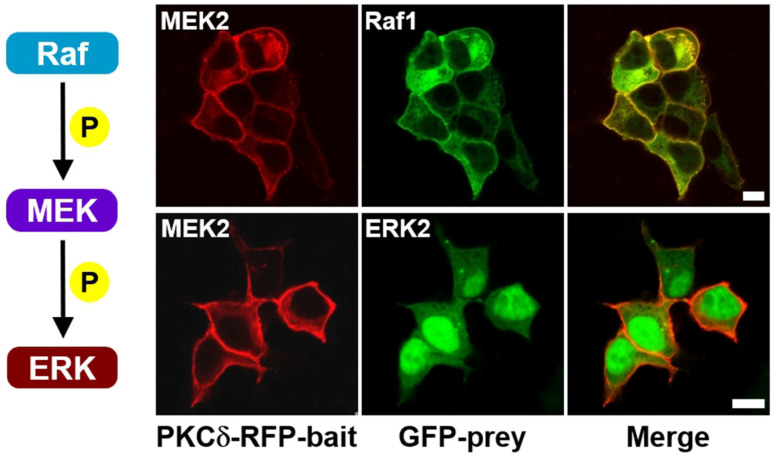
Confocal images of the Raf–MEK–ERK signaling cascade in living cells without exogenous expression of scaffold proteins. HEK-293T cells were co-transfected with PKCδ–mRFP–MEK2 (bait) and either eGFP–Raf1 or eGFP–ERK2 (prey). Transiently co-transfected cells were serum-starved for 16–18 h in serum-free DMEM before stimulation with EGF. After induction with 100 ng/mL of EGF for 5 min, the cells were treated with PMA (final concentration 1 μM) in serum-free DMEM. Raf1 was co-translocated with MEK2 to the plasma membrane due to the translocation property of PKCδ (**top row**); in comparison, only MEK2 protein from the MEK2/ERK2 pair was translocated to the plasma membrane (**bottom row**). The images were acquired 3–5 min after PMA treatment. The scale bar is 10 μm.

First, we imaged the Raf–MEK–ERK signaling cascade in living cells without scaffold proteins. The fusion constructs PKCδ–mRFP–MEK2 and eGFP–Raf1 (or ERK2) were prepared and co-expressed in cells. In the absence of serum stimulation, only MEK protein was translocated to the plasma membrane; in comparison, neither Raf nor ERK protein co-translocated to the plasma membrane (Figure S1A,B, top row). Conversely, in the presence of EGF, a serum stimulus induced the ERK pathway, resulting in the co-translocation of Raf to the plasma membrane (Figure 1, top row, and Figure S1A, bottom row). However, the ERK protein was not co-translocated to the plasma membrane (Figure 1, bottom row, and Figure S1B, bottom row). The results of the co-immunoprecipitation assay also confirmed these results (Figure S2). In studies using co-immunoprecipitation (Co-IP) to investigate Raf/MEK interaction [5], C-Raf (Raf1)/MEK interaction required exogenous overexpression. Regarding B-Raf/MEK interaction, it was found that when MEK was exogenously overexpressed, binding to endogenous B-Raf was observed independent of KSR1. However, under endogenous conditions, binding of MEK to B-Raf was only detected in cells expressing KSR1. Despite exogenous overexpression, we were unable to detect MEK/ERK binding. Consistent with the results of previous studies, our results imply that overexpression of scaffold proteins is essential for visualizing MEK/ERK interaction in this experimental context.

**Figure 2:**
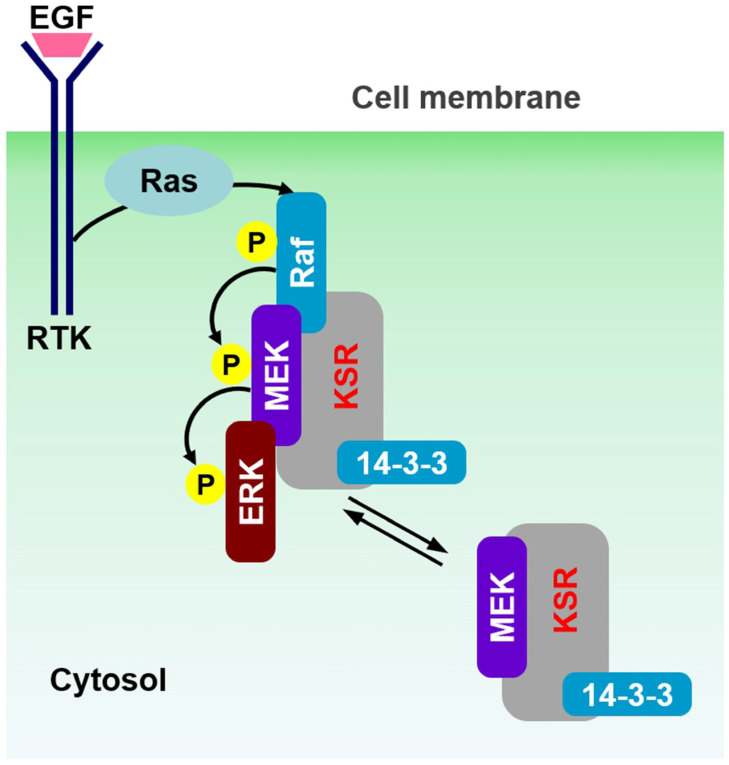
A schematic representation of the Raf–MEK–ERK signaling cascade, including the KSR scaffold protein in cells. In quiescent cells, the KSR scaffold protein is typically cytoplasmic and constitutively bound to MEK and 14–3-3. Upon receptor tyrosine kinase (RTK) stimulation, signaling cells initially utilize KSR to enhance signal transmission from Raf to MEK by facilitating Raf/MEK interaction. Subsequently, KSR attenuates signaling by docking activated ERK. Overall, KSR regulates the intensity and duration of Raf–MEK–ERK signaling cascade activation.

**Figure 3:**
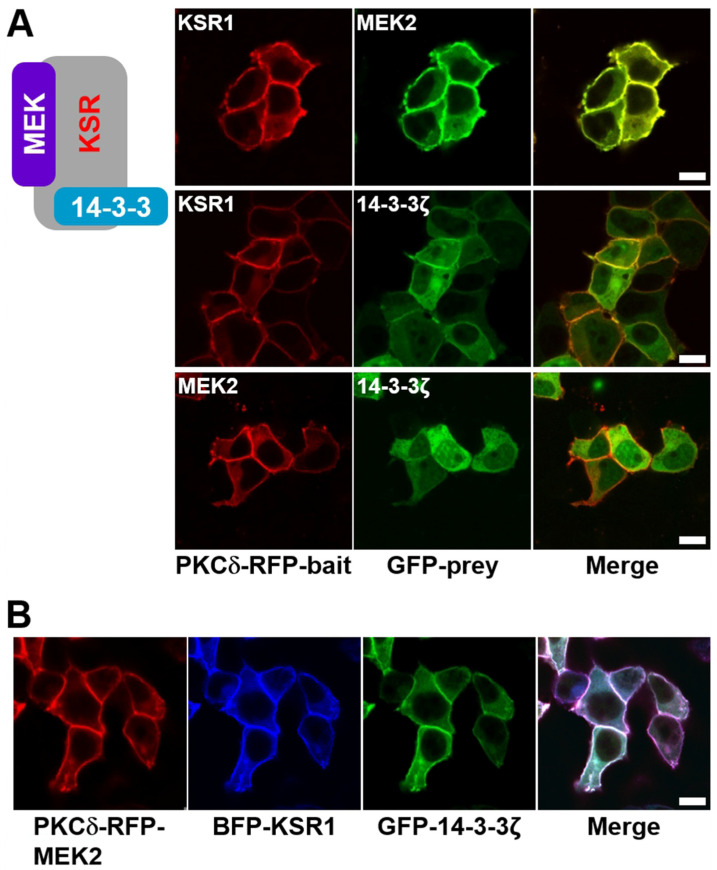
Confocal images of the KSR1/MEK2/14-3-3ζ ternary protein complex in quiescent cells. (**A**) HEK-293T cells were transiently co-transfected with the following protein pairs: PKCδ–mRFP–KSR1/eGFP–MEK2, PKCδ–mRFP–KSR1/eGFP–14-3-3ζ, and PKCδ–mRFP–MEK2/eGFP–14-3-3ζ. After PMA treatment, KSR1 and MEK2 were co-translocated to the plasma membrane, with evidence of constitutive interaction between them (**top row**); KSR1 and 14-3-3ζ were also co-translocated to the plasma membrane, with their interaction shown (**middle row**)*;* and MEK2 was translocated alone to the plasma membrane, showing no interaction with 14-3-3ζ (**bottom row**). (**B**) HEK-293T cells were transiently co-transfected with PKCδ–mRFP–MEK2, TagBFP–KSR1, and eGFP–14-3-3ζ. The ternary protein complex, MEK2/KSR1/14-3-3ζ, was co-translocated to the plasma membrane after PMA treatment. The images were acquired 3-5 min after PMA treatment. The scale bar is 10 μm.

Most recently, scaffold proteins have been implicated in the regulation of signaling cascades in mammals. The primary role of scaffold proteins is to assemble proteins essential for specific signal transduction events into close proximity, thereby facilitating their interaction. KSR1 functions as a scaffold protein in the Raf–MEK–ERK signaling cascade, independent of its kinase activity. Thus, it was demonstrated that KSR1 is associated with MEK in quiescent cells, but not with Raf or ERK kinases. KSR1 can bind all kinase members of the ERK pathway; however, whereas MEK is associated constitutively, Raf and ERK might bind in a stimulus-dependent manner. Furthermore, Raf and the inactive KSR/MEK complex are localized in a quiescent cell in the cytosolic region. Upon stimulation and Ras activation, Ras activation recruits Raf to the plasma membrane. The KSR/MEK complex also moves to the membrane, where it interacts with Raf, leading to enhanced MEK activation. Simultaneously, ERKs are recruited to the activated complex, facilitating their phosphorylation and activation (Figure 2) [4,5,6,7,8,9,10,11]. KSR scaffold protein is usually cytoplasmic and constitutively bound to MEK and 14-3-3 protein (Figure 2) [7]. We prepared the PKCδ–mRFP–KSR1 and eGFP–MEK2 (or 14-3-3ζ) fusion constructs and co-expressed them in cells. Figure 3A shows that the KSR scaffold protein binds to MEK and 14-3-3 protein, respectively (Figure S3A,B), and there is no direct interaction between MEK and 14-3-3 protein (Figure 3A, bottom row and Figure S3C). Furthermore, the KSR scaffold protein-containing ternary complex, MEK2/KSR1/14-3-3ζ, was co-translocated to the plasma membrane (Figure 3B). The KSR/MEK protein complex can translocate to the plasma membrane in response to RTK and Ras activation. We imaged overexpressed MEK/KSR/14-3-3 cells after EGF treatment. However, we did not observe a change in the subcellular localization of the KSR/MEK protein complex under our experimental conditions (Figure S4).

**Figure 4:**
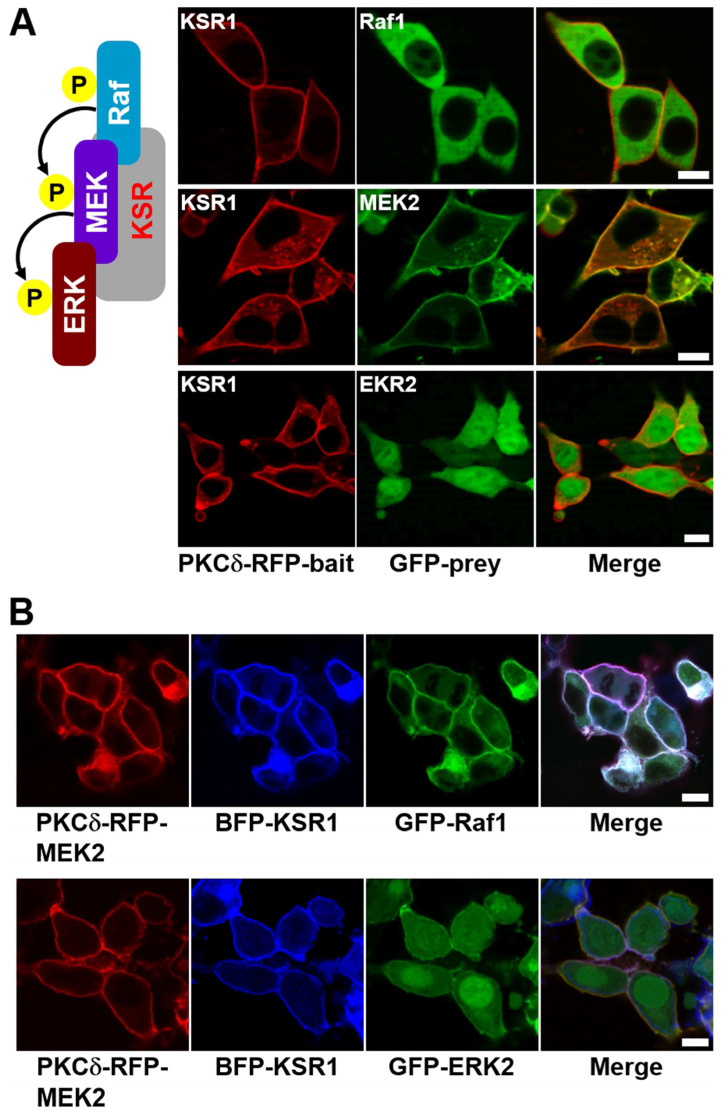
Confocal images of the Raf–MEK–ERK signaling cascade in living cells with KSR1 scaffold protein. (**A**) HEK-293T cells were transiently co-transfected with PKCδ–mRFP–KSR1 and each of eGFP–Raf1, eGFP–MEK2, and eGFP–ERK2. The cells were then serum-starved, stimulated with EGF, and treated with PMA. KSR1 translocation to the plasma membrane was observed, with or without interaction with Raf1, MEK2, or ERK2, as indicated (**top**, **middle**, and **bottom rows**, respectively). (**B**) HEK-293T cells were transiently co-transfected with either PKCδ–mRFP–MEK2/TagBFP–KSR1/eGFP–Raf1 or PKCδ–mRFP–MEK2/TagBFP–KSR1/eGFP–ERK2. The cells were then serum-starved, stimulated with EGF, and treated with PMA. The ternary protein complexes, both Raf1/KSR1/MEK2 and MEK2/KSR1/ERK2, were co-translocated to the plasma membrane. The images were acquired 3–5 min after PMA treatment. The scale bar is 10 μm.

Next, we investigated the role of KSR1 in the Raf–MEK–ERK signaling cascade to determine whether it was required for the observed MEK/ERK interaction under our experimental conditions. We first imaged the interactions between KSR1 and both Raf1 and ERK2. No direct interaction was observed between KSR1 and either Raf1 or ERK2 (Figure 4A, top and bottom row, respectively, and Figure S5A,C); however, the addition of MEK2 protein facilitated interactions between KSR and both Raf1 and ERK2 (Figure 4B). The results of GST pull-down assays also confirmed these results (Figure S6). Additionally, in the absence of serum stimuli, only the KSR/MEK protein complex was co-translocated to the plasma membrane (Figure S7, second row). Together, these results confirm that KSR1 acts as a scaffold protein, facilitating MEK/ERK interaction and thereby enhancing signal transduction in signaling cells.

## 3. Materials and Methods

### 3.1. Construction of Bait/Prey Expression Vectors

The wild-type genes, PKCδ (BC043350), Raf1 (BC018119), MEK2 (BC018645), ERK2 (M64300), KSR1 (BC167812), and 14-3-3ζ (BC003623), were obtained from Open Biosystems (Thermo Fisher Scientific Inc., Waltham, MA, USA). mRFP (DQ903889) was kindly supplied by MeDiscove Inc. (Daejeon, Republic of Korea) and was amplified via the polymerase chain reaction (PCR).

To produce the pPKCδ-mRFP-C3 plasmid, the eGFP gene was removed from the peGFP-C3 (Clontech Inc., San Jose, CA, USA). The PCR-amplified mRFP gene was then inserted into the modified vector using AgeI and BglII restriction sites. Subsequently, the PCR-amplified PKCδ gene was cloned into pmRFP-C3 using NheI and AgeI restriction sites, as previously reported [22]. Lastly, PKCδ–mRFP–bait (MEK2 and KSR1) expression vectors were constructed using pPKC-mRFP-C3. The eGFP–prey expression vectors, including each of the PCR-amplified Raf1, MEK2, ERK2, and 14-3-3ζ genes, were cloned into either peGFP-C3 or peGFP-N1. For the expression vectors of TagBFP–KSR1 and GST–KRS1, the PCR-amplified KSR1 gene was cloned into pTagBFP-N1 (Evrogen, Moscow, Russia) and pEBG (Addgene, Watertown, MA, USA), respectively.

### 3.2. Co-Expression of the Bait/Prey Protein Pair in Cultured Cells

HEK-293T cells were grown on 25 mm round coverslips (Paul Marienfeld GmbH & Co. KG, Lauda-Königshofen, Germany) in a 6-well culture plate to 50–70% confluence. Transient co-transfection of the desired bait/prey protein pair (Raf1/MEK2, MEK2/ERK2, KSR1/Raf1, KSR1/MEK2, KSR1/ERK2, KSR1/14-3-3ζ, MEK2/KSR1/14-3-3ζ, Raf1/KSR1/MEK2, and MEK2/KSR1/ERK2, etc.) was conducted using a TurboFect (Thermo Fisher Scientific Inc. Waltham, MA, USA) according to the manufacturer’s standard protocol.

### 3.3. Confocal Imaging

Transiently co-transfected cells were serum-starved for 16–18 h in serum-free DMEM before stimulation with EGF (Invitrogen, Waltham, MA, USA). The serum-starved cells on round coverslips were mounted on a homemade perfusion chamber, connected to a temperature controller set at 37 °C. The cells were washed with serum-free DMEM (without phenol red) pre-warmed to 37 °C and then treated with 100 ng/mL of EGF for 5 min. Sequential images of the same cell were collected at 10 sec intervals using a laser-scanning confocal microscope (LSM 800, Carl Zeiss, Oberkochen, Germany) with a C-Apochromat 40x/1.2 water immersion lens at the Korea Basic Science Institute (Ochang, Republic of Korea). During imaging, PMA was added to the chamber (the total concentration of PMA in the serum-free medium was 1 μM, with the PMA being obtained from Sigma-Aldrich, St. Louis, MO, USA).

### 3.4. Co-Immunoprecipitation (Co-IP) and GST Pull-Down Assays

For Co-IP, HEK-293T cells were transiently co-transfected with either mRFP–Raf1/eGFP–MEK2 or mRFP–MEK2/eGFP–ERK2. Transiently co-transfected cells were serum-starved overnight in serum-free DMEM and then stimulated with 100 ng/mL of EGF for 5 min. The cells were washed once with cold PBS and resuspended in NP-40 lysis buffer for 30 min at 4 °C. The cells were disrupted and centrifuged to remove insoluble debris. Lysates were incubated for 3 h with the GFP antibody (Santa Cruz Biotechnology, Dallas, TX, USA), followed by protein A-Sepharose beads (Cytiva, Amersham, England) for 1 h at 4 °C. For GST pull-down, HEK-293T cells were transiently co-transfected with either GST–KSR1/mRFP–Raf1/eGFP–MEK2 or GST–KSR1/mRFP–MEK2/eGFP–ERK2. Transiently co-transfected cells were serum-starved overnight and then stimulated with EGF. The cells were washed once with cold PBS and resuspended in NP-40 lysis buffer for 30 min at 4 °C. The cells were disrupted and centrifuged to remove insoluble debris. Lysates were incubated with glutathione Sepharose 4B beads (Cytiva, Amersham, England) at 4 °C for 3 h. Immune complexes were washed and then analyzed via immunoblotting.

## 4. Conclusions

In this work, we developed a translocation-based cellular assay to visualize the Raf–MEK–ERK signaling cascade in living cells. This assay enables the co-translocation of specific interacting protein pairs, such as Raf, MEK, and ERK, from the cytoplasm to the plasma membrane in response to an exogenous stimulus, with the KSR1 scaffold protein regulating cascade activation. We successfully visualized direct Raf1/MEK2 interaction (Figure 1 top row) and the KSR1-containing ternary complex (Raf1/MEK2/KSR1) (Figure 4B top row). However, the interaction between MEK and ERK, specifically direct MEK2/ERK2 interaction, was not observed (Figure 1, bottom row). We successfully observed MEK2/ERK2 interaction with the KSR1 scaffold protein (Figure 4B, bottom row). We are confident that this method will serve as a valuable platform for the image analysis of signaling cascades within living systems. Furthermore, translocation-based cellular assays generally require minimal construction optimization and are durable, rapid, and versatile while also ensuring a high signal-to-noise ratio. Based on the above results, we believe that our method provides a useful imaging analysis platform for studying binding events, such as protein−protein interactions and their inhibition using a compound library in living cells.

## Data Availability

Data are contained within the article and Supplementary Materials.

## Supplementary Materials

The following supporting information can be downloaded at: https://www.mdpi.com/article/10.3390/ijms251910587/s1.

## References

[1] Zhang W., Liu H.T. (2002). MAPK signal pathways in the regulation of cell proliferation in mammalian cells. Cell Res..

[2] Ma M., Bordignon P., Dotto G.P., Pelet S. (2020). Visualizing cellular heterogeneity by quantifying the dynamics of MAPK activity in live mammalian cells with synthetic fluorescent biosensors. Heliyon.

[3] Ullah R., Yiu Q., Snell A.H., Wan L. (2022). RAF–MEK–ERK pathway in cancer evolution and treatment. Semin. Cancer Biol..

[4] Martin-Vega A., Cobb M.H. (2023). Navigation the ERK1/2 MAPK cascade. Biomolecules.

[5] McKay M.M., Ritt D.A., Morrison D.K. (2009). Signaling dynamics of the KSR1 scaffold complex. Proc. Natl. Acad. Sci. USA.

[6] Meister M., Tomasovic A., Banninb A., Tikkanen R. (2013). Mitogen-activated protein (MAP) kinase scaffolding proteins: A recount. Int. J. Mol. Sci..

[7] Kolch W. (2005). Coordination ERK/MAPK signaling through scaffolds and inhibitors. Nat. Rev. Mol. Cell Biol..

[8] Parvathaneni S., Li Z., Sacks D.B. (2021). Calmodulin influences MAPK signaling by binding KSR1. J. Biol. Chem..

[9] Shaul Y.D., Seger R. (2007). The MEK/ERK cascade: From signaling specificity to diverse functions. Biochim. Biophys. Acta.

[10] Liu Z., Krstic A., Neve A., Casalou C., Rauch N., Wynne K., Cassidy H., McCann A., Kavanagh E., McCann B. (2023). Kinase suppressor of RAS 1 (KSR1) maintains the transformed phenotype of BRAFV600E mutant human melanoma cells. Int. J. Mol. Sci..

[11] Maloney R.C., Zhang M., Liu Y., Jang H., Nussinov R. (2022). The mechanism of activation of MEK1 by B–Raf and KSR1. Cell. Mol. Life Sci..

[12] Lyons A.C., Mehta S., Zhang J. (2023). Fluorescent biosensors illuminate the spatial regulation of cell signaling across scales. Biochem. J..

[13] Newman R.H., Fosbrink M.D., Zhang J. (2011). Genetically encodable fluorescent biosensors for tracking signaling dynamics in living cells. Chem. Rev..

[14] Tomida T. (2015). Visualization of the spatial and temporal dynamics of MAPK signaling using fluorescence imaging techniques. J. Physiol. Sci..

[15] Peterson A.F., Ingram K., Huang E.J., Parksong J., McKenney C., Bever G.S., Regot S. (2022). Systematic analysis of the MAPK signaling network reveals MAP3K-driven control of cell fate. Cell Syst..

[16] Weeks R., Mehta S., Zhang J. (2024). Genetically encodable biosensors for Ras activity. RSC Chem. Biol..

[17] Zaver S.A., Johnson C.J., Berndt A., Simpson C.L. (2023). Live imaging with genetically encoded physiological sensors and optogenetic tools. J. Investig. Dermatol..

[18] Heydorn A., Lundholt B.K., Praestegaard M., Pagliaro L. (2006). Protein translocation assays: Key tools for accessing new biological information with high-throughput microscopy. Methods Enaymol..

[19] Piljić A., Schultz C. (2008). Analysis of protein complex hierarchy in living cells. ACS Chem. Biol..

[20] Mentrup T., Häsler R., Fluhrer R., Saftig P., Schröder B. (2015). A cell-based assay reveals nuclear translocation of intracellular domains released by SPPL proteases. Traffic.

[21] Jain R., Saini D.K. (2013). Imaging cellular signalling: Many ‘moving tales’ in MAP kinase odyssey. Curr. Sci..

[22] Lee K.B., Hwang J.M., Choi I.S., Rho J., Choi J.S., Kim G.H., Kim S.I., Kim S., Lee Z.W. (2011). Direct monitoring of the inhibition of protein-protein interaction in cells by translocation of PKCδ fusion proteins. Angew. Chem. Int. Ed..

[23] Hwang J.M., Lee K.B., Choi J.S., Rho J., Kim G.H., Kim S.I., Kim S., Lee Z.W. (2011). Novel technology for protein-protein interaction-based targeted drug discovery. J. Anal. Sci. Technol..

[24] Wang Q.J., Bhattacharyya D., Garfield S., Nacro K., Marquez V.E., Blumberg P.M. (1999). Differential localization of protein kinase C δ by phorbol esters and related compounds using a fusion protein with green fluorescent protein. J. Biol. Chem..

[25] Lim P.S., Sutton C.R., Rao S. (2015). Protein kinase C in the immune system: From signalling to chromatin regulation. Immunology.

[26] Kawano T., Inokuchi J., Eto M., Murata M., Kang J.H. (2022). Protein kinase C (PKC) isozymes as diagnostic and prognostic biomarkers and therapeutic targets for cancer. Cancers.

