# Supplementary material for: Imaging the Raf–MEK–ERK Signaling Cascade in Living Cells
Source: Int J Mol Sci. 2024 Oct 1;25(19):10587. doi: 10.3390/ijms251910587 (PMC11477372; doi:10.3390/ijms251910587)
Supplement: Supplementary file 1 [file ijms-25-10587-s001.zip › ijms-3200511-supplementary.pdf]

Supplementary Materials

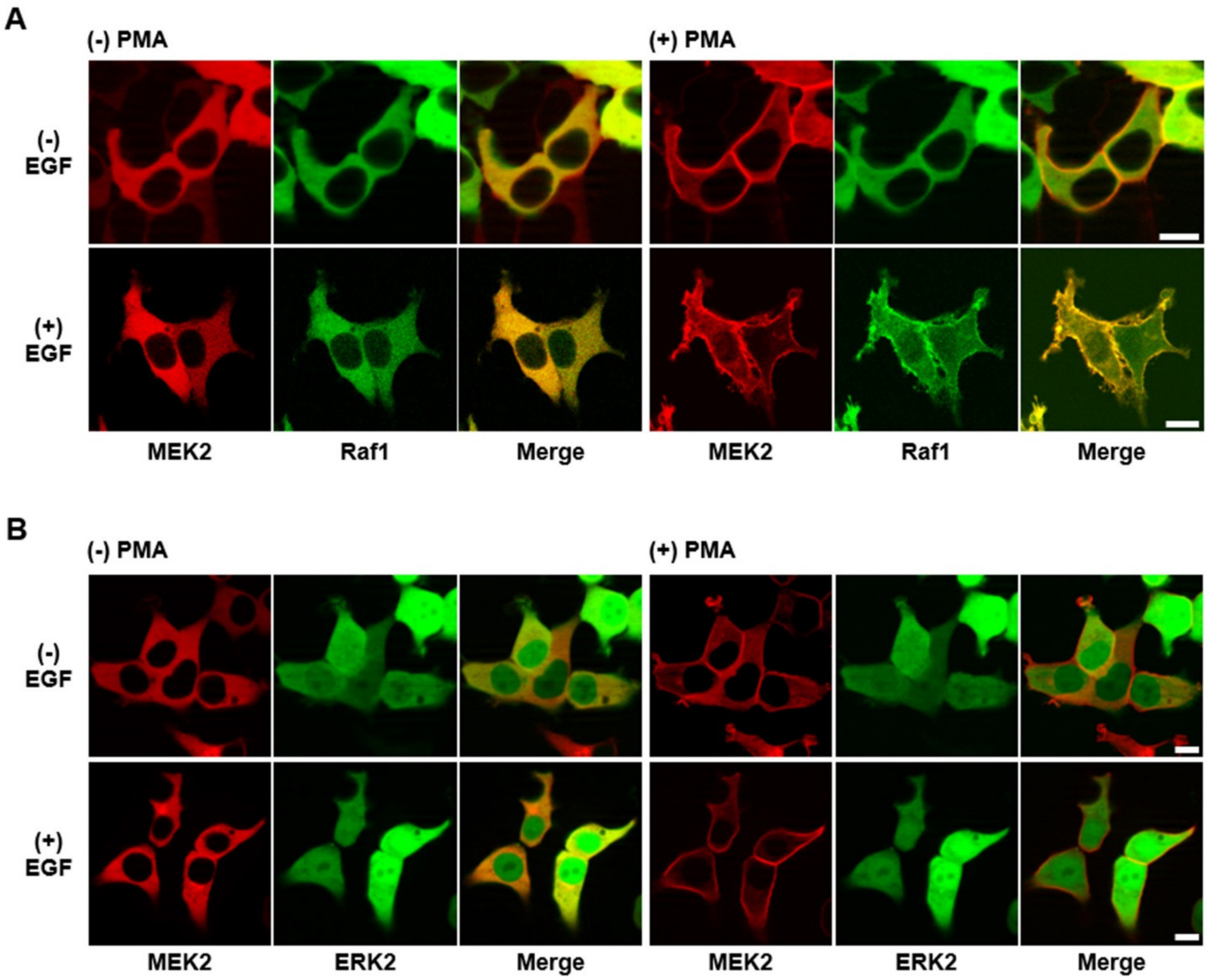

Figure S1. Confocal images of the RAF–MEK–ERK signaling cascade in living cells without exogenous expression of scaffold proteins. HEK-293T cells were co-transfected with PKCδ–mRFP–MEK2 (bait) and either eGFP–Raf1 or eGFP–ERK2 (prey). Transiently co-transfected cells were serum-starved for 16–18 h in serum-free DMEM before stimulation with EGF. After induction with 100 ng/mL of EGF for 5 min, cells were treated with PMA (final concentration 1 mM) in serum-free DMEM. (A) Without serum stimulation, only MEK protein was translocated to the plasma membrane (top row); with EGF stimulation, Raf1 and MEK2 were co-translocated to the plasma membrane (bottom row). (B) Interaction between MEK2 and ERK2 was not observed under either condition. The images were acquired 3-5 min after PMA treatment. The scale bar is 10 μm.

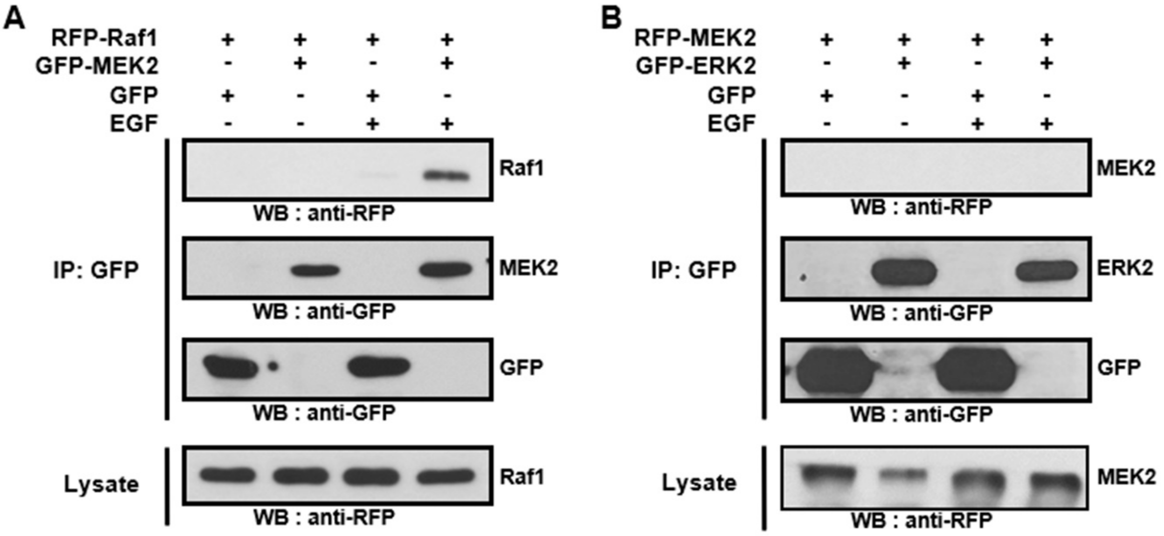

Figure S2. HEK-293T cells were transiently co-transfected with either mRFP–Raf1/eGFP–MEK2 (A) or mRFP–MEK2/eGFP–ERK2 (B). Transiently co-transfected cells were serum-starved overnight in serum-free DMEM and then stimulated with EGF. The co-immunoprecipitation assay showed an interaction between Raf1 and MEK2 (A) but not between MEK2 and ERK2 (B).

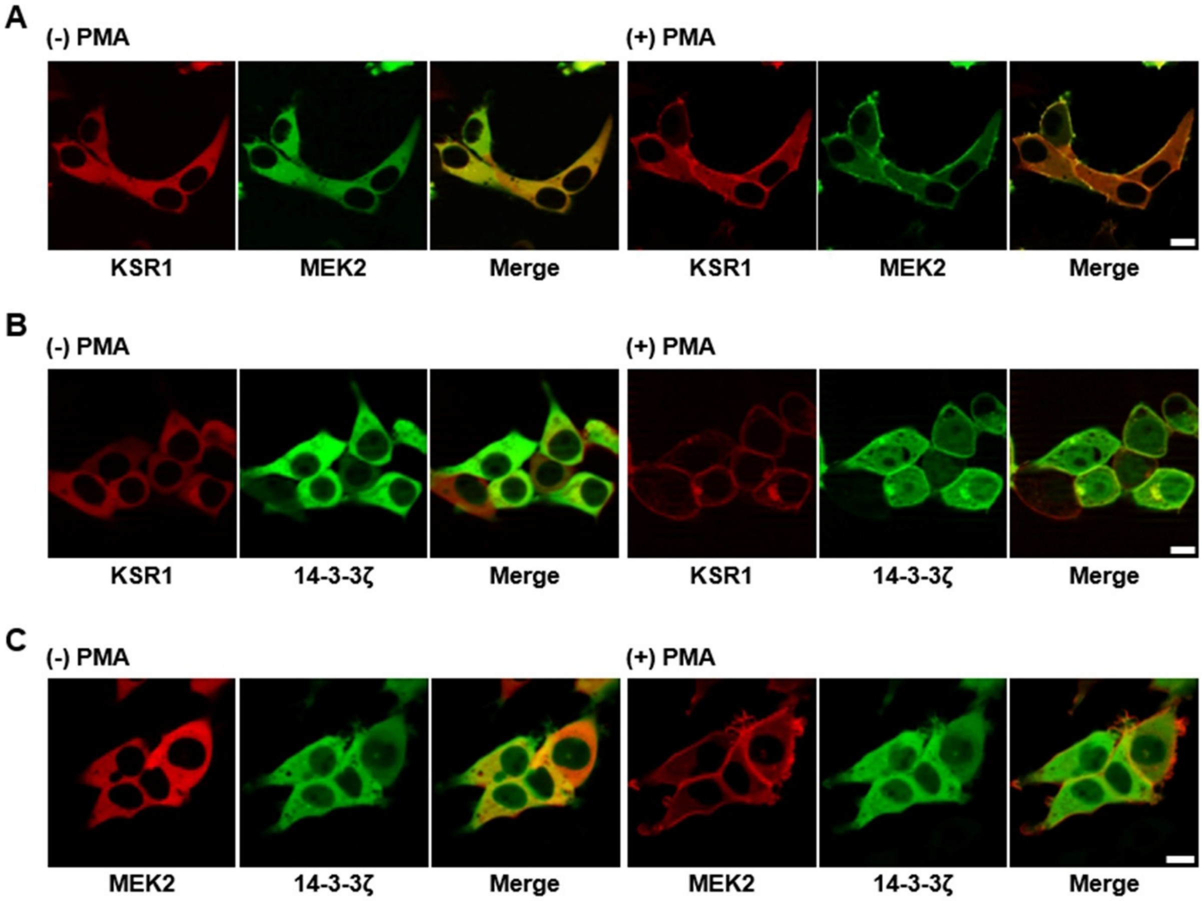

Figure S3. HEK-293T cells were transiently co-transfected with the following protein pairs: PKCδ–mRFP–KSR1/eGFP–MEK2 (A), PKCδ–mRFP–KSR1/eGFP–14-3-3ζ (B), and PKCδ–mRFP–MEK2/eGFP–14-3-3ζ (C). After PMA treatment, (A) KSR1 and MEK2 were co-translocated to the plasma membrane, exhibiting a constitutive interaction; (B) KSR1 and 14-3-3ζ were co-translocated to the plasma membrane, showing an interaction between them; and (C) no interaction was observed between MEK2 and 14-3-3ζ. The images were acquired 3-5 min after PMA treatment. The scale bar is 10 μm.

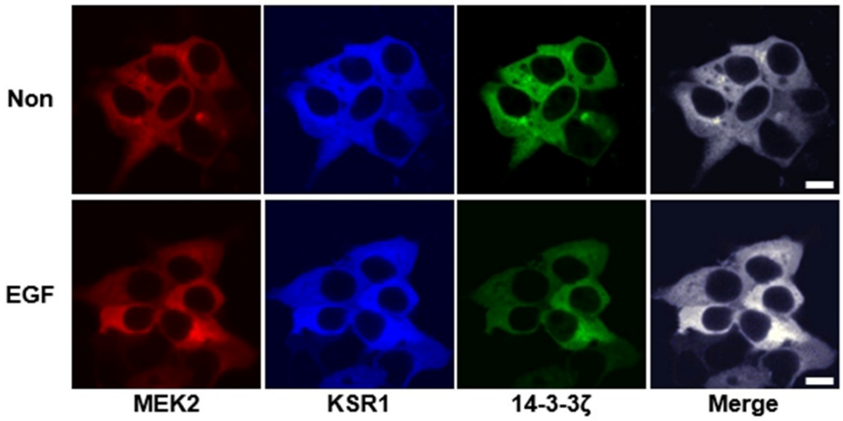

Figure S4. HEK-293T cells were transiently co-transfected with PKCδ–mRFP–MEK2/TagBFP–KSR1/eGFP–14-3-3ζ. Cells overexpressing MEK/KSR/14-3-3 were imaged without (top row) or with EGF stimulation (bottom row). The scale bar is 10 μm.

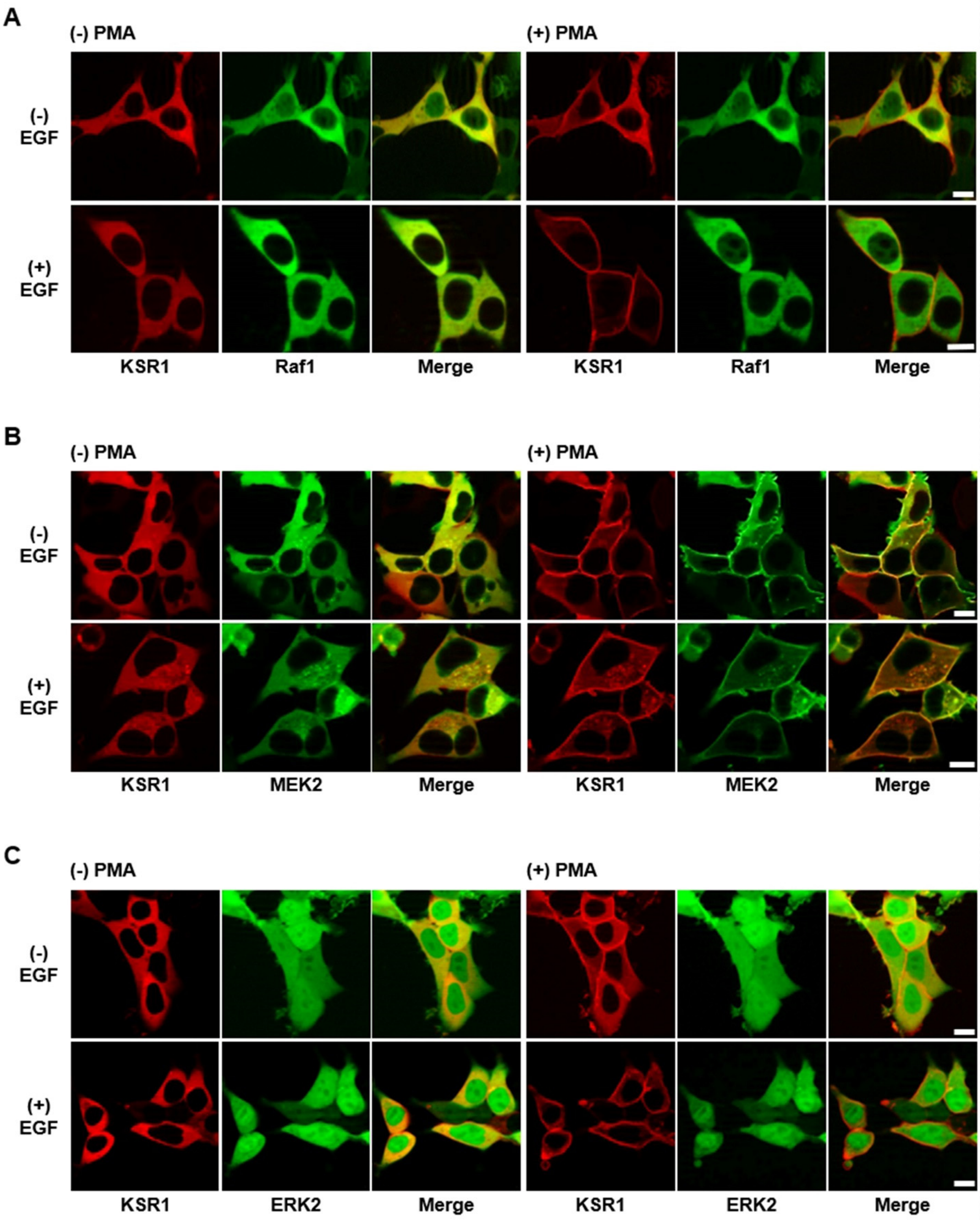

Figure S5. HEK-293T cells were transiently co-transfected with PKCδ–mRFP–KSR1 and each of eGFP–Raf1, eGFP–MEK2, and eGFP–ERK2. The cells were then serum-starved, stimulated with EGF, and treated with PMA. KSR1 translocation to the plasma membrane was observed with or without serum stimulation (A, B, and C), only the MEK protein was bound to KSR1 under either condition (B). The images were acquired 3-5 min after PMA treatment. The scale bar is 10 μm.

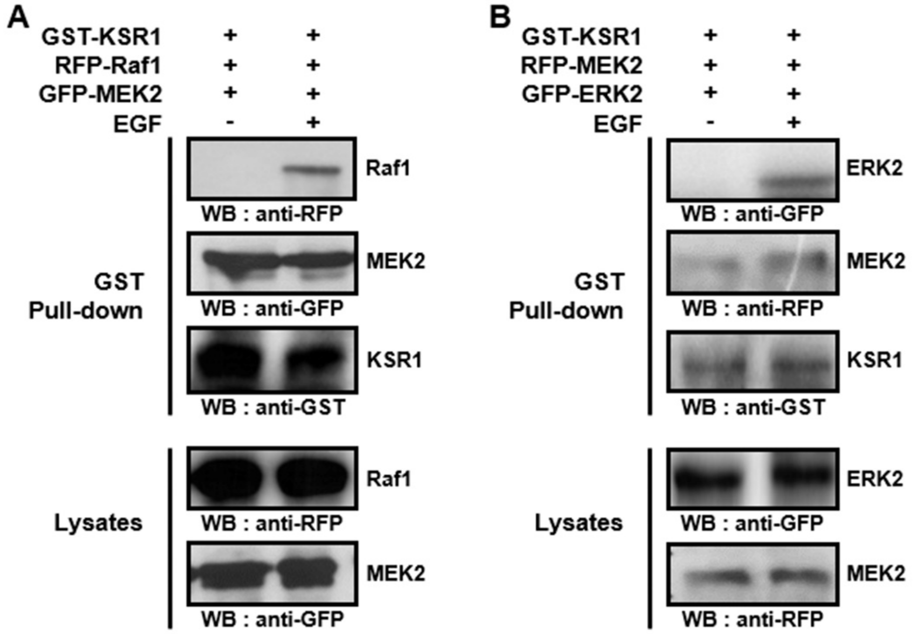

Figure S6. HEK-293T cells were transiently co-transfected with either GST–KSR1/mRFP–Raf1/eGFP–MEK2 (A) or GST–KSR1/mRFP–MEK2/eGFP–ERK2 (B). The co-transfected cells were serum-starved overnight in serum-free DMEM and then stimulated with EGF. GST pull-down assays showed that KSR1 interacted with both Raf1 and MEK2 (A), and with both MEK2 and ERK2 (B) following EGF stimulation.

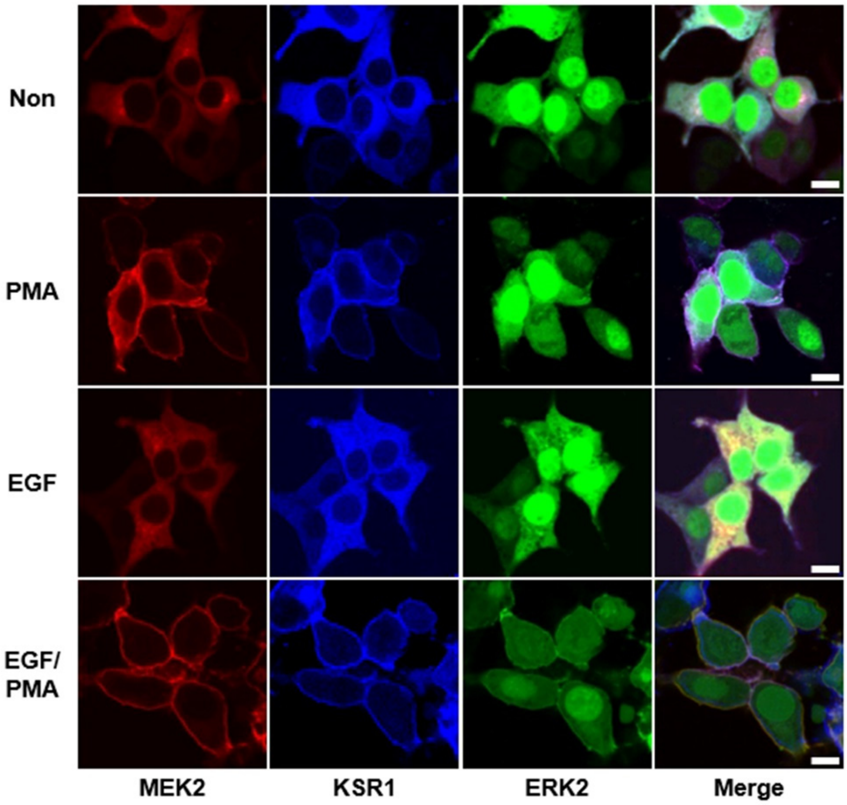

Figure S7. HEK-293T cells were transiently co-transfected with PKCδ–mRFP–MEK2, TagBFP–KSR1, and eGFP–ERK2. Cells overexpressing MEK/KSR/ERK were imaged without (second row) or with EGF stimulation (bottom row). In the absence of serum stimuli, only the KSR/MEK protein complex was co-translocated to the plasma membrane (second row). The ternary protein complex, MEK2/KSR1/ERK2, was co-translocated to the plasma membrane in the presence of serum stimuli (bottom row). The images were acquired 3-5 min after PMA treatment (second and bottom rows). The scale bar is 10 µm.
